# Predictive Value of Dynamic Cerebral Autoregulation Assessment in Surgical Management of Patients with High-Grade Carotid Artery Stenosis

**DOI:** 10.3389/fphys.2017.00872

**Published:** 2017-11-02

**Authors:** Vladimir B. Semenyutin, Gregory A. Asaturyan, Anna A. Nikiforova, Vugar A. Aliev, Grigory K. Panuntsev, Vadim B. Iblyaminov, Alexander V. Savello, Andreas Patzak

**Affiliations:** ^1^Laboratory of Brain Circulation Pathology, Federal Almazov Medical Research Center, Saint-Petersburg, Russia; ^2^Department of Neurosurgery, Municipal Hospital of Saint Martyr Elizabeth, Saint-Petersburg, Russia; ^3^Johannes-Mueller Institute of Physiology University Hospital Charite, Humboldt University of Berlin, Berlin, Germany

**Keywords:** carotid artery stenosis, cerebral autoregulation, cerebral hemodynamics, surgery/endarterectomy, transcranial Doppler

## Abstract

Dynamic cerebral autoregulation (DCA) capacity along with the degree of internal carotid artery (ICA) stenosis and characteristics of the plaque can also play an important role in selection of appropriate treatment strategy. This study aims to classify the patients with severe ICA stenosis according to preoperative state of DCA and to assess its dynamics after surgery. Thirty-five patients with severe ICA stenosis having different clinical type of disease underwent reconstructive surgery. DCA was assessed with transfer function analysis (TFA) by calculating phase shift (PS) between Mayer waves of blood flow velocity (BFV) and blood pressure (BP) before and after operation. In 18 cases, regardless of clinical type, preoperative PS on ipsilateral side was within the normal range and did not change considerably after surgery. In other 17 cases preoperative PS was reliably lower both in patients with symptomatic and asymptomatic stenosis. Surgical reconstruction led to restoration of impaired DCA evidenced by significant increase of PS in postoperative period. Our data suggest that regardless clinical type of disease various state of DCA may be present in patients with severe ICA stenosis. This finding can contribute to establishing the optimal treatment strategy, and first of all for asymptomatic patients. Patients with compromised DCA should be considered as ones with higher risk of stroke and first candidates for reconstructive surgery.

## Introduction

Numerous randomized clinical trials demonstrated the benefit of surgical management of severe symptomatic internal carotid artery (ICA) stenosis for the treatment and prevention of ipsilateral cerebral ischemic events (European Carotid Surgery Trialist's Collaborative Group, 1991; North American Symptomatic Carotid Endarterectomy Trial Collaborators, 1991). As for the asymptomatic patients the current efficacy of surgical procedures vs. optimal medical therapy is still deemed to be debatable (Halliday et al., 2004; Abbott and Nicolaides, 2015). The choice of treatment is firstly based on assessment of the degree of ICA stenosis and embologenecity of the atherosclerotic plaque (Rothweell et al., 2003; Gupta et al., 2015). The other important determinants are multiple co-morbidity risk factors that inevitably increase the rate of periprocedural cerebral or cardiac complications and can lead to poor outcome after intervention (Fanning et al., 2014). But even in “good-risk” population it is still difficult to identify the patients who are particularly at high threat of stroke and would most benefit from carotid reconstruction. Along with traditional imaging markers numerous studies emphasize significant predictive value of cerebrovascular reserve that might help in clarifying the patients for surgical or medical treatment options (Marshall et al., 2003; King et al., 2011; Gupta et al., 2012). Reduced cerebrovascular reserve indicates that the arteriolar vasodilation activity is not able to maintain stable blood flow properly in the region of cerebral hypoperfusion. It mostly depends on the degree of stenosis and the level of collateral circulation in the same region (Reinhard et al., 2003a; Cheng et al., 2012). Gupta et al. (2012) in their systematic meta-analysis found strong association between vasomotor reactivity impairment in response to acetazolamide or CO_2_ inspiration stimulus and risk of stroke or transient ischemic attack in both high-grade ICA stenosis or occlusion as well as in symptomatic and asymptomatic patients. Similar results were obtained evaluating dynamic cerebral autoregulation (DCA) based on assessment of blood flow velocity (BFV) changes in middle cerebral arteries (MCA) in response to slow oscillations of systemic arterial blood pressure (BP) using different testing modalities in the time and frequency domain (Hu et al., 1999; Reinhard et al., 2003a; Haubrich et al., 2004; Tang et al., 2008). The detailed methodology of such approach and it's validity for non-invasive assessment of DCA has been previously reported by many researches (Diehl et al., 1995; Hu et al., 1999; Haubrich et al., 2004; Reinhard et al., 2004). It based on the concept that cerebral regulatory system functions as a high-pass filter that means that high-frequency fluctuations (cardiac, 0.65–1.4 Hz and respiratory, 0.15–0.65 Hz) of BP are normally passing through BFV unimpeded while low-frequency oscillations (within the range of systemic Mayer waves, 0.08–0.12 Hz) are dampened. As a result, slow waves of BFV do not occur simultaneously to similar waves of BP but with a time delay. Various pathological states of the brain (intracranial hypertension, cerebral vasospasm, hemorrhage, ischemia) due to the head injury, hydrocephalus, aneurysm rupture, occlusion of the main cerebral arteries are usually accompanied by DCA impairment leading to reducing the time delay even up to zero (Lang et al., 2001; Müller et al., 2003; Kim et al., 2015; Castro et al., 2017). Previous studies demonstrated clear postoperative normalization of DCA in severe ICA stenosis that is generally impaired ipsilaterally prior to surgical intervention (Reinhard et al., 2004; Tang et al., 2008). This finding is regarded as an important predictive indicator of stroke risk and poor functional outcome while choosing proper treatment strategy (Reinhard et al., 2003a; King et al., 2011; Gupta et al., 2012). However, it remains unclear whether DCA could be unaffected or slightly impaired in severe ICA stenosis. Moreover, there is no consensus nowadays which way of treatment modality would be more effective for both asymptomatic and symptomatic patients. Some authors insist on preventive operation in preserved DCA to avoid ischemic stroke, while others prefer using “wait-and-see” attitude and adhere to medical therapy (Silvestrini et al., 2000; Reinhard et al., 2003a; Isozaki et al., 2010). This clinical study aims to classify the patients with severe ICA stenosis according to preoperative state of DCA and to assess its dynamics after surgical intervention in relation to postoperative outcome.

## Materials and methods

### Subjects and management

The design of the study was approved by Local ethical standards Committee of Federal Almazov Medical Research Center in accordance with the Helsinki Declaration of 1975 (and as revised in 1983) and performed after obtaining written informed consent from the subjects or their legal representatives. No animal studies or human experiments were conducted as part of this work.

We prospectively examined a cohort of 42 consecutive patients with severe ICA stenosis (83 ± 9%) who were admitted to neurovascular department for surgical management. Thirty-five patients were enrolled in the study. Collected data included clinical type of disease, degree of stenosis, results of neuroimaging, type of surgical treatment, and co-morbidity risk factors (Table 1). Clinical assessment of all patients, based on full neurological examination by an independent neurologist, was ranged according to modified Rankin scale (mRs) (Banks and Marotta, 2007). The patients underwent ultrasound assessment of precerebral arteries with standard criteria by a single specialist using Vivid E ultrasound system (GE, USA) equipped with multi-frequency linear transducer (4–12 MHz). Plaque structure was assessed in B-mode and defined according to its echogenicity and embologenicity based on Gray–Weale's classification (Gray-Weale et al., 1988). The degree of carotid stenosis and postoperative results were documented in compliance with North American Symptomatic Carotid Endarterectomy Trial (NASCET) method. (North American Symptomatic Carotid Endarterectomy Trial Collaborators, 1991) Along with ultrasound imaging standardized computed tomography (CT) angiography with multislice CT scanner Brilliance 64 (Philips, Netherlands) was also administered to all patients preoperatively to assess severity of stenosis with NASCET-type method. Transcranial Doppler (TCD) ultrasound by Multidop X TCD (DWL, Sipplingen, Germany) with 2 MHz transducers was assigned to measure mean BFV bilaterally in the basal cerebral arteries and to evaluate collateral flow capacity. Indications for interventions—by carotid endarterectomy (CEA) or carotid balloon angioplasty with stenting (CAS)—were determined by interdisciplinary consensus of neurosurgeon and interventional neuroradiologist based on analysis of the individual risk factors of each modality. Type of CEA was dependent on carotid artery anatomy as well as the plaque extension, collateral circulation efficacy, and surgeon's technique preferences. Patients subjected to CAS received dual antiplatelet therapy with Clopidogrel (75 mg) and Aspirin (100 mg) before the procedure and 6 months thereafter. Stenting was performed using self-expanding nitinol stent (Precise, Cordis, USA) and emboli-protection device (Angioguard Embolic Capture Guidewire, Cordis, USA). Symptomatic patients underwent surgical intervention 81 ± 51 (M ± m) days after last ischemic episode. Exclusion criteria were poor acoustic temporal bone windows for TCD ultrasonography (*n* = 3), individual intolerance of examination (*n* = 2), and severe cardiac failure or arrhythmia (*n* = 2). Neurological and ultrasound examinations were implemented 3 days prior to operation as well as at discharge (7–10 days after intervention).

**Table 1:** Characteristics of the control group and groups of patients with asymptomatic and symptomatic carotid artery stenosis.

**Variable**	**Control group (*n* = 15)**	**Asymptomatic group (*n* = 24)**	**Symptomatic group (*n* = 11)**
Age (year) (M ±m)	55 ± 7	65 ± 10	70 ± 6
Gender (male/female), n	8/7	15/9	10/1
The degree of stenosis (%)(M ± m)	–	82 ± 10	83 ± 10
Type of atherosclerotic plaque according gray-weale scale, n			
I	–	3	1
II	–	20	7
III	–	1	2
IV	–	–	1
Type of operation CEA/CAS, n	–	12/12	5/6
Modified Rankin scale, preoperative/postoperative (M ± m)	–	0.5 ± 0.5/0.5 ± 0.3	1.2 ± 0.8/1.0 ± 0.5
Hypertension, n	–	23	10
Diabetus mellitus, n	–	3	1
Ischemic heart disease, n	–	17	9
Baseline mean BP (mm Hg) (M ± m)	89 ± 12	97 ± 10	92 ± 11
Baseline BFV in MCA (cm/s) ipsilateral/contralateral (M ± m)	68 ± 18^*^	69 ± 37/76 ± 20	63 ± 12/67 ± 23
Baseline PS (rad) ipsilateral/contralateral (M ± m)	1.1 ± 0.2^*^	0.5 ± 0.3/0.8 ± 0.5	0.4 ± 0.2/1.0 ± 0.5

CEA, Carotid endarterectomy; CAS, carotid balloon angioplasty with stenting; BP, blood pressure; BFV, blood flow velocity; MCA, middle cerebral artery; PS, phase shift;

* *values for the right middle cerebral artery in control group*.

Fifteen age-matched healthy subjects with no history of stroke or heart attack as well as other vascular disease were also recruited into the study for comparative analysis. Supposing that they have an intact DCA on both sides all measurements were performed only in right MCA.

### Dynamic cerebral autoregulation assessment

DCA measurements were performed in a supine position with 30° elevation of the upper body while the subjects were breathing at a rate of 6/min. End-tidal CO_2_ partial pressure measured with capnograph Tidalwave (Novametrix, Philips Respironics, Netherlands) was corresponded to normocapnia (36–38 mm Hg). BFV in both MCA was insonated through the temporal bone window at a depth of 50–60 mm with 2 MHz probe fixed on a head-frame. BP was registered continuously via a servo-controlled finger photoplethysmography (CNAP Monitor 500 HD, CNSystems Medizintechnik AG, Austria) with the subject's hand position at heart level. BP recording was transmitted through analog input channel of Multidop X system block into a monitoring module of DWL-software. After establishing stable synchronized signals of both BFV and BP the servo-calibration option of CNAP was turned off and data over 5 min were recorded simultaneously at a sampling frequency 50 Hz. Data segments were visually inspected and edited for artifacts and ectopy. Only steady-state data were digitized and stored on a hard disk in universal ASC files of DWL-software for further off-line DCA assessment.

### Frequency and transfer function analysis

Transfer function analysis (TFA) based on Welch algorithm is a suitable method to calculate the specific parameters of DCA phenomenon (Schytz et al., 2010; Claassen et al., 2016). For this power spectra of BP and BFV and their cross-spectrum were estimated by transforming the time series of BP and BFV with Fast Fourier transformation to the frequency domain. The data were analyzed in windows containing data segments of 512 points (280 s), with 50% overlap. Prior to TFA mean values were subtracted from the data and Hanning anti-leakage window with 15-points width of data window was used to minimize spectral leakage. The respective periodograms were smoothed to obtain the spectrum power. The spectral amplitude of BP and BFV derived from spectral power, phase shift (PS), and coherence coefficient were calculated in specifically selected range of systemic Mayer waves (M-waves). This frequency band commonly reflects periodic fluctuations of BP and is most informative for the assessment of myogenic component of cerebrovascular response comparing with high frequency oscillations. According to the high-pass filter model pressure-flow relation between the input (BP) and output (BFV) signals is characterized by positive PS (0.8–1.2 rad). In case of impaired autoregulation passing capacity of the filter for slow waves increases, and therefore PS between both parameters reduces, even up to zero. Coherence between 0 and 1 reflects the linear relation between BP and BFV. To ensure the highest reliability of PS-values for further statistical analyses they were estimated only at frequencies which exhibited coherence >0.6 and maximum of M-waves' amplitude in BP. TFA was performed with commercially available data acquisition software “Statistica for Windows 7” (StatSoft Inc., Oklahoma, USA) in “Time Series and Forecasting” Module.

### Statistical analysis

Descriptive statistics were used to describe demographic characteristics, as well as patients' clinical, angiographic, ultrasound, and TFA data. Patients were dichotomized into symptomatic and asymptomatic groups with impaired and unaffected DCA. Inter-group and intra-group differences were tested using non-parametric tests (χ^2^ with Fisher exact, univariate Mann–Witney, Kolmogorov–Smirnov) for categorical data and Student *t*-test for normally distributed (Shapiro–Wilk test) continuous variables. The correlation between clinical course of ICA stenosis and autoregulatory parameters (PS, coherency, M-wave amplitude) were assessed by Spearman's rank coefficient method. A paired Student *t*-test was used to estimate the significance of changes after carotid reconstruction in all groups. Parametric data are expressed as mean ± *SD*, and values of *p* < 0.05 are considered statistically significant. Statistical analysis was performed using “Statistica for Windows 7” software (StatSoft Inc., Oklahoma, USA).

## Results

In total, 35 patients (25 men and 10 womens, aged 66 ± 9) with severe ICA stenosis were enrolled in this study. The demographic and clinical characteristics of included healthy controls and patients are presented in Table 1.

### Patient characteristics

Twenty four (69%) patients had asymptomatic course of disease with no previous history of ischemic events but mild cognitive disorders while 11 (31%) others were symptomatic. Among 11 patients with symptomatic ICA stenosis prior to admission transient ischemic attacks occurred in two cases and ischemic stroke—in nine cases. The severity of stroke according to National Institutes of Health Stroke Scale (NIHSS) (Brott et al., 1989) varied from 3 to up 11 and was in an average 5.7 ± 2.7 points. In two patients the symptoms of stroke regressed within 7 days. Other 7 patients had partial regression of symptoms within 1 month after stroke and suffered of persistent neurologic disorders on admission.

Surgical treatment of stenosis by CEA was performed in 17 (49%) subjects and by CAS—in 18 (51%) subjects. Early postoperative complications after CEA were registered in three patients: one had subcutaneous hematoma of the neck that required a revision of the wound and two—temporary palsy of hypoglossal nerve with complete regression of symptoms at the discharge. In two patients remote moderate restenosis was noted 6 months after intervention. These complications did not compromise cerebral circulation as well as not worsen functional outcome and thus were not considered as exclusion criteria. Neither stroke nor transient ischemic attack as well as hyperperfusion syndrome occurred postoperatively. Co-morbidity risk factors were noted in 33 (94%) out of 35 patients and included arterial hypertension (94%), coronary heart disease (74%), diabetus mellitus (11%). No differences between the groups were seen with respect to clinical variables except that co-morbidity factors as well as type II of atherosclerotic plaque by Gray-Weale's classification (Gray-Weale et al., 1988) within this study were noted more frequently in asymptomatic patients.

### Systemic and cerebral hemodynamics characteristics

There was no significant difference in the baseline values of BP and BFV in MCA on ipsilateral side between the two clinical groups (Table 1). Moreover, no strict correlation was observed between preoperative results of DCA assessment and the clinical course of the disease, plaque type, gender, comorbid factors, type of surgical intervention, initial functional state of patients in accordance with the mRs.

PS findings on the side of disorder showed insignificant tend toward DCA impairment in symptomatic group. In most individuals with symptomatic ICA stenosis (*n* = 7; 64%) DCA was impaired (PS < 0.8 rad) while in prevailing number of patients with asymptomatic clinical course (*n* = 14; 58%) DCA indices were within normal limits (PS ≥ 0.8 rad).

**Figure 1:**
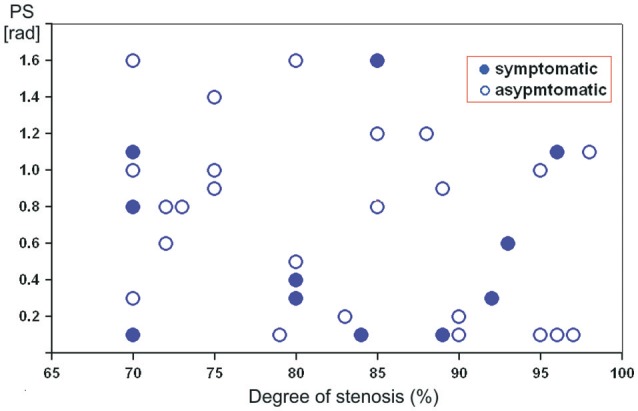
Scatterplot showing the relation between preoperative phase shift values (in radians) on the affected side and the degree of severe internal carotid artery stenosis in asymptomatic and symptomatic patients.

Figure 1 illustrates the relationship between DCA on ipsilateral side and various degree of severe ICA stenosis. A tend toward a negative correlation between PS and stenosis severity was revealed. However, in the same degree of stenosis reduced or normal PS-values were registered in both clinical groups.

Table 2 contains the data of systemic and cerebral hemodynamics on affected side including DCA indices before and after reconstructive operations in different subgroups of asymptomatic and symptomatic ICA stenosis with regard to the baseline state of DCA (impaired vs. unimpaired autoregulation).

**Table 2:** BP, BFV in MCA, and PS (M ± m) on ipsilateral side in different groups of carotid stenosis before/after reconstructive surgery.

	**Impaired DCA**		**Unimpaired DCA**	
	**Asymptomatic (*n* = 10)**	**Symptomatic (*n* = 7)**	**Asymptomatic (*n* = 14)**	**Symptomatic (*n* = 4)**
BP (mmHg)	92 ± 7/91 ± 11^*^	91 ± 20/81 ± 12^*^	98 ± 15/93 ± 14^*^	90 ± 8/89 ± 3^*^
BFV (cm/s)	78 ± 21/76 ± 16^*^	63 ± 9/67 ± 13^*^	64 ± 10/67 ± 13^*^	63 ± 15/65 ± 19^*^
PS (rad)	0.2 ± 0.2/0.9 ± 0.5^**^	0.3 ± 0.2/0.9 ± 0.3^**^	1.1 ± 0.3/0.9 ± 0.3^*^	1.3 ± 0.5/1.3 ± 0.5^*^

BP, blood pressure; BFV, blood flow velocity; MCA, middle cerebral artery; PS, phase shift;

* p > 0.4;

** *p < 0.001*.

According to TFA 10 asymptomatic and seven symptomatic patients from the study showed dysfunction of high-pass filter for M-waves, suggesting that DCA on the side of carotid stenosis was impaired. Despite the normal baseline values of BFV in both MCA in this subgroup, PS on ipsilateral side varied between 0.1 and 0.6 rad. Reconstructive surgery resulted in increasing of PS up to 0.6–1.0 rad for asymptomatic and to 0.5–1.2 rad for symptomatic patients. No significant postoperative BP and BFV changes were evident. Functional improvement according to mRs represented by partial regression of cerebral and/or focal symptoms after surgery was registered in only two symptomatic patients (from 2 to 1 point) and in three asymptomatic patients as well (from 1 to 0).

**Figure 2:**
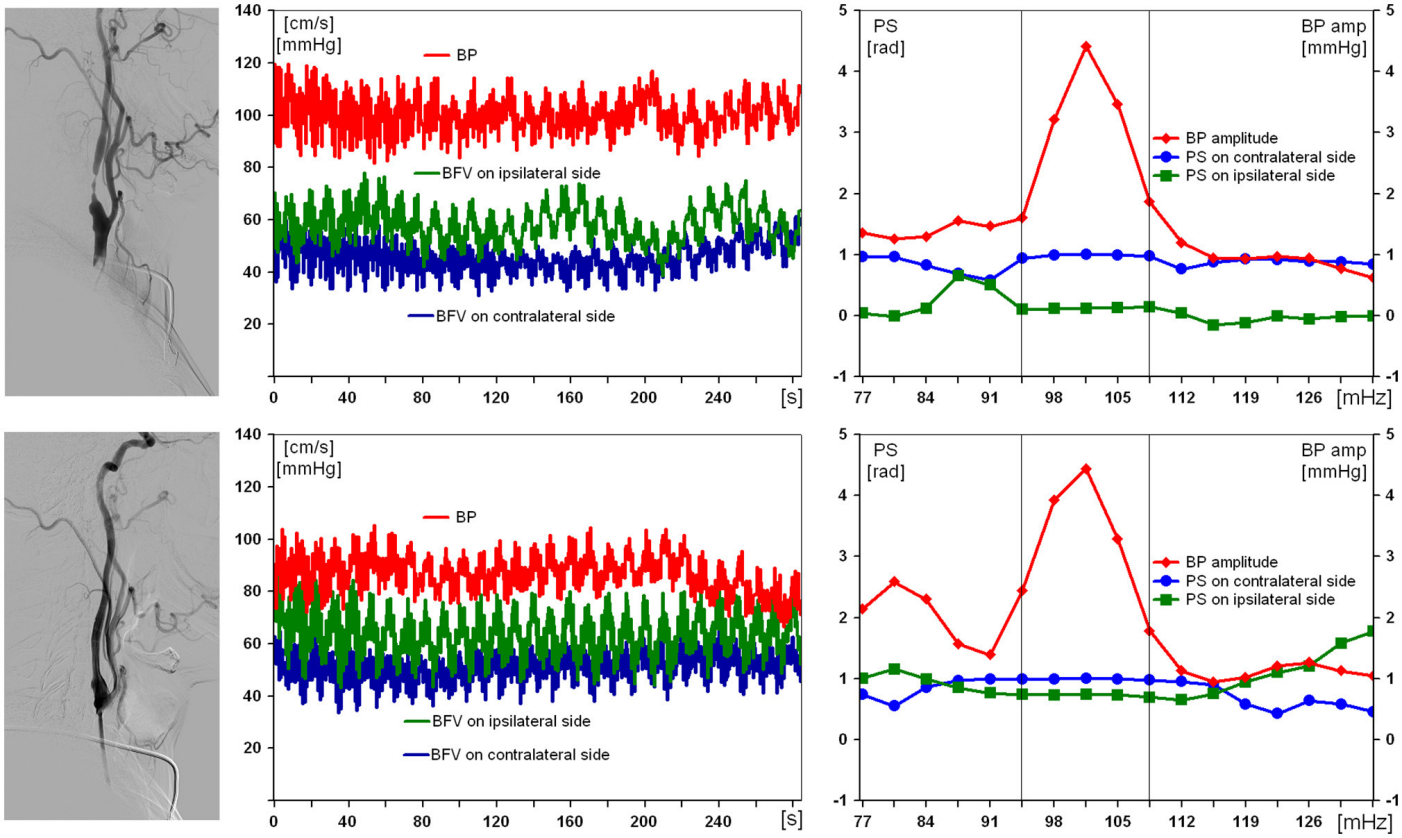
Results of examination of a 59-year-old man with 97% asymptomatic right carotid artery stenosis and impaired dynamic autoregulation on ipsilateral side. From left to right: carotid angiogram, multichannel monitoring of blood pressure (BP) and blood flow velocity in middle cerebral arteries over 5 min during compulsory breathing at a rate 6/min, phase shift (PS) and BP-amplitude spectra within the range of Mayer waves. From top to bottom: before and 7 days after carotid balloon angioplasty with stenting. The selected in-box area in spectral analysis comprises the significant harmonic components of PS to determine its average value.

Results obtained from the examination of the patient with a severe right ICA stenosis and impaired DCA are presented in Figure 2. Suffering with mild arterial hypertension he had no ischemic history, thus the severe ICA stenosis was defined as “incidental finding.” Preoperative values of BFV in both MCA were within normal limits, with slight asymmetry, but only pulsatility index was reduced up to 0.58 in ipsilateral MCA. PS on the affected side before carotid stenting was 0.1 rad. On the 7th day after endovascular reconstruction PS restored up to 0.9 rad, and no postoperative neurological complication was observed.

As for the rest of 14 patients with asymptomatic and four patients with symptomatic severe carotid artery stenosis, TFA testified reliably good functional ability of cerebrovascular reserve. Thus, these patients were included into the subgroup with unimpaired DCA. The values of PS before and after operation were within normal limits and varied between 0.8 and 1.6 rad. There was no significant difference of BFV in both MCA between two subgroups. After surgical treatment no cerebral and systemic hemodynamic changes were noted. Unreliable functional improvement according mRs was registered only in three cases: two with asymptomatic and one with symptomatic ICA stenosis (from 1 to 0 point).

**Figure 3:**
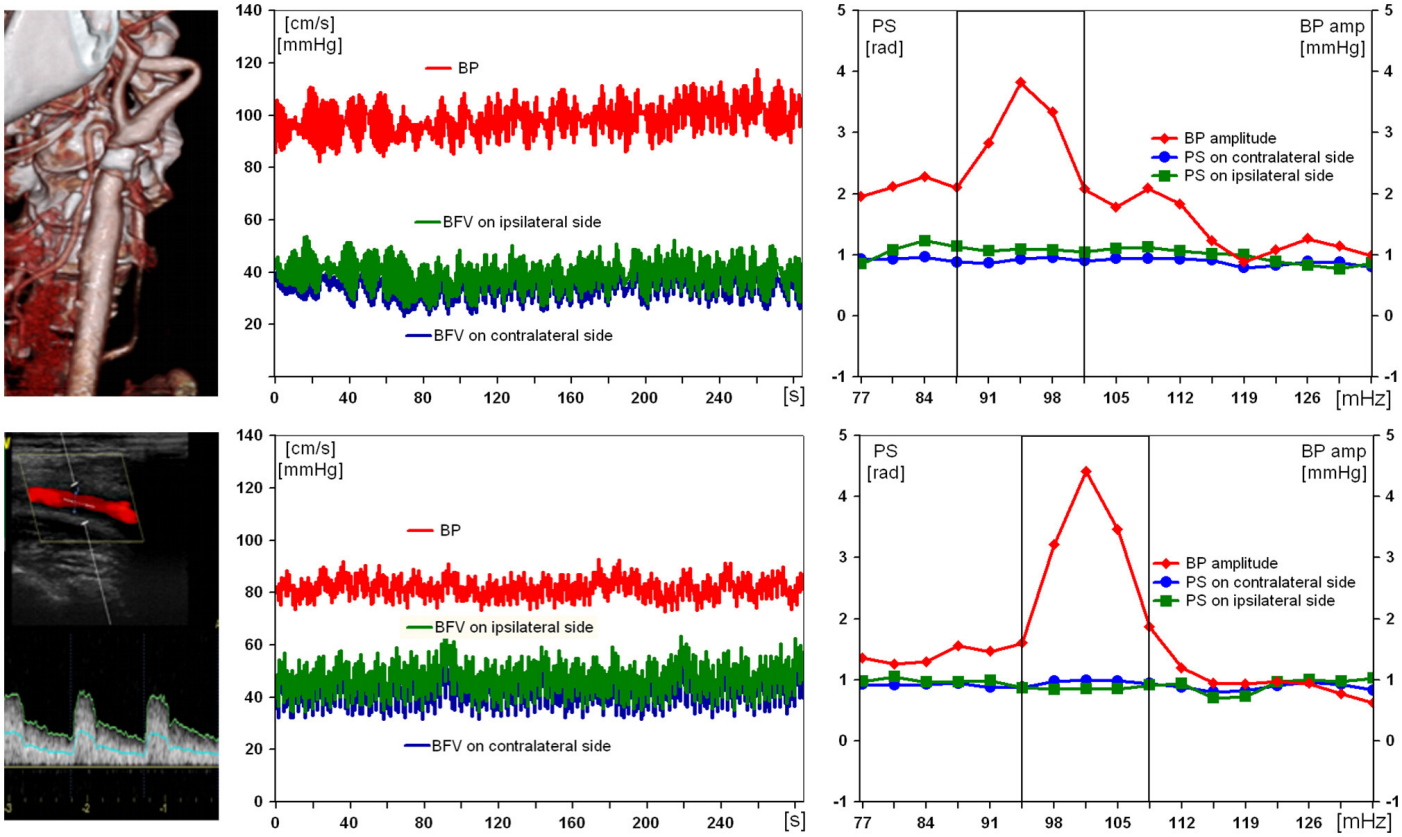
Results of examination of a 69-year-old man with 89% symptomatic right carotid artery stenosis and preserved dynamic autoregulation on ipsilateral side. From left to right: computed tomography angiogram and duplex scanning of carotid artery, multichannel monitoring of blood pressure (BP) and blood flow velocity in middle cerebral arteries over 5 min during compulsory breathing at a rate 6/min, phase shift (PS) and BP-amplitude spectra within the range of Mayer waves. From top to bottom: before and 10 days after carotid endarterectomy.

Figure 3 illustrates the results of examination of the patient with a severe ICA stenosis and unimpaired DCA. Being positive for acute stroke in ipsilateral MCA, the patient had normal preoperative values of PS (1.1 rad) on affected side, as well as of BFV and BP (47 cm/s and 90 mm Hg, respectively). On the 10th day after CEA no considerable changes of all hemodynamic parameters were noted.

## Discussion

An impaired DCA represents one of the key pathophysiological mechanisms triggering brain ischemic lesions (Reinhard et al., 2003a; Schytz et al., 2010; Gupta et al., 2012). Considering that the severe stenosis may significantly reduce cerebral perfusion pressure which could reach its lower limit for autoregulation, compensatory vasodilation under these conditions and in response to further natural, even slight, reductions of BP may be inadequate and would not be able to protect the brain from hemodynamic ischemic events. On the other hand, restoration of cerebral perfusion pressure up to normal values after reconstructive surgery may contribute to development of hyperperfusion syndrome since microcirculatory vessels in the presence of persistent critical vasodilation lack the ability to timely provide an adequate vasoconstriction.

In our research, reconstructive surgery in both clinical groups with impaired DCA led to evidently postoperative improving of autoregulation (increase of PS) on the affected side, which is consistent with the results obtained in numerous currently available studies (Hartl et al., 1994; Reinhard et al., 2004; Tang et al., 2008). The exceptions were only two patients with ICA restenosis, which might be responsible for no changes in PS postoperatively. However, not all patients with restored DCA had clinical improvement when assessing functional results with mRs in a remote period after surgical treatment (from 6 months to 1 year after operation).

Along with that it should also be noted that in some patients with symptomatic/asymptomatic severe ICA stenosis DCA was not impaired at all before planning surgery. None of these patients in the study showed reliable changes of PS, BFV, or BP, and their clinical condition according to mRs remained unaffected in both early and remote postoperative period. Such “a good behavior” of DCA despite the severity of stenosis which is apparently associated with sufficient quality of collateral circulation and microcirculatory vasodilation capacity was also mentioned by other researchers (Hartl et al., 1994; Marshall et al., 2003; Reinhard et al., 2003a). Therefore, it may be concluded that high-grade ICA stenosis in case of unimpaired DCA on ipsilateral side should not be absolutely considered as a risk-factor that fatefully can lead to cerebral infarction. In this regard, we suppose that the dynamic follow-up with assignment of optimal medical therapy would be more preferable for this category of patients. This is of particular concern for individuals with asymptomatic severe ICA stenosis as the indications for surgical treatment of them are still controversial and require further in-depth study (Halliday et al., 2004; Paraskevas et al., 2015). The 2 year risk of ipsilateral stroke events in the natural history of the disease in cohort of asymptomatic patients who are receiving optimal medical therapy (<1%) is quite lower than the procedural (30-day) risk of probable postoperative complications for CEA (1.4%) and CAS (2.5%) (Spence et al., 2014; Hart and Ng, 2015).

The lack of clinical improvement in 12 patients with postoperative restored DCA may be due to the fact that the initial functional state in these patients before surgery did not substantially affect their everyday social activity. In symptomatic patients slight limitation of regular life activity (Table 1) could be due to persistent local structural changes in an infarction zone, which despite the restoration of perfusion and DCA in the region of the basal intracranial artery on affected side, nevertheless have appeared to be irreversible (paresis, a dysphasia) after surgery. In this regard, it is the DCA index that could be a decisive predictive factor when choosing surgical treatment modality. Another reason can be the presence of concomitant cardiovascular pathology (ischemic heart disease, arrhythmia), which determined moderate impairment of the patient's functional activity before and after the surgery.

It should be noted that the control group was somewhat younger than the group of patients with ICA stenosis (Table 1). At the same time, as it follows from Table 2, even in older patients from subgroup with unimpaired DCA on affected side, mean values of PS did not differ from the normal values of subjects from the control group. In this regard, we believe that if the control group was adjusted according to age with a group of patients, it would not change the findings we obtained. In addition, a similar difference of the age between the control group and the group of patients is found in other works (Diehl et al., 1995; Hu et al., 1999; Haubrich et al., 2004).

As for the cases of high-grade ICA stenosis manifesting only with cognitive disorders, the choice of treatment strategy is conventionally based on additional prognostic factors (Nicolaides et al., 2010). Therein, some authors pay great attention to characteristics of atherosclerotic plaque, and first of all its embologenicity, while others emphasize the importance of cognitive disorders' severity, as well as the presence of co-morbidity risk factors and historic ischemic events in other vascular regions (contralateral carotid artery, vertebral arteries; Abbott et al., 2005; Nicolaides et al., 2010; Demarin et al., 2012; Gupta et al., 2015). According to our results we suggest that the baseline state of DCA in this group of patients is equally important factor and in some cases it may play the key role in choice of treatment. From this point of view, patients with impaired DCA are likely to have the highest risk of stroke, thus, they ought to be considered as the first candidates for surgery.

The last statement is certainly true for the patients with symptomatic severe ICA stenosis, since according to literature data a disturbance of cerebrovascular reserve is revealed in most cases of ipsilateral stroke. Undoubtedly, they have to be subjected to the surgical treatment as soon as possible after onset of the disease (Vandamme and Limet, 2005). It is noteworthy, that 4 out of 11 patients from the same cohort in our study had normal indices of DCA on admission and were characterized with mild neurological deficit after acute event and early regression of the symptoms (minor stroke) despite the severity of stenosis. At the same time, other seven patients had persistent neurologic disorders after stroke and DCA on ipsilateral side was in poor condition prior to surgery.

The question remains open about atherosclerotic plaque's instability, which increases the risk of stroke due to microembolia. But its identification often has some difficulties. Besides, detection of microembolia based on TCD monitoring does not allow reliably to specify the source of these emboli and the etiology of the stroke (atherotrombotic, cardioembolic, etc.). This may complicate predicting stroke due to the plaque instability and determining treatment strategy (Gupta et al., 2015). Additionally, some data suggest that the use of optimal medical therapy reduces the verified microemboli on TCD from 12.6 to 3.7% (Nicolaides et al., 2010; Gupta et al., 2015). Patients with asymptomatic clinical course or microembolia that are not related to carotid stenosis therefore require conservative rather than surgical treatment (Spence et al., 2014).

In our study, a majority of patients had stable homo-, hyperechogenic plaques of type I-II by the Gray-Weale scale (Table 1). Apparently, that is why we did not find any significant differences of PS in both clinical groups of ICA stenosis in dependence with plaque type. It may be assumed that DCA in patients with unstable heterogeneous plaque (type III–IV) will be impaired to a greater degree, since in addition to hemodynamic factors (decrease of perfusion pressure distal to ICA stenosis), the role of thrombembolic factors in stroke development significantly increases. But this assumption does not contradict our conclusions, as patients with symptomatic stenosis and disturbed DCA are considered by us as the first candidates for surgical intervention. The study of this hypothesis is undoubtedly of scientific and practical interest, and is the subject of a separate research study.

We did not also find reliable differences of PS and clinical type of disease with respect to gender, comorbid factors, type of surgical intervention, initial functional state of patients in accordance with the mRs. Talking about the functional state, it should be added that patients with symptomatic stenosis were examined and operated in the early recovery period −81 ± 51 (M ± m) after stroke onset. In this regard, the functional state of these patients in a majority of cases was satisfactory in (1.2 ± 0.8 mRs) at the time of admission for surgical treatment. Moreover, in some cases it did not differ from the functional state of the patients with asymptomatic ICA stenosis. Petersen et al. demonstrated the restoration of impaired DCA within 1 week after large-vessel acute ischemic stroke in middle cerebral artery territory (Petersen et al., 2015). The study included 28 patients (NIHSS −12 ± 6.5), among which in nine cases the etiology of stroke was large-vessel atherosclerosis (ICA stenosis), but the percentage of ICA narrowing was <70%. Our data show that even in 11 patients with critical symptomatic ICA stenosis DCA can also be restored to normal values, but month later after onset. Also noteworthy that severity of stroke (NIHSS −5.7 ± 2.7) was quite lower than in Petersen study and only in four cases we found normal values of DCA on affected side at the time of admission. No doubt, further larger prospective study is needed to analyze the changes in DCA depending on the severity of stroke and its etiology, as well as the functional ability of the patients with high-grade ICA stenosis at different periods of the stroke.

The absence of reliable differences between asymptomatic and symptomatic groups with respect to the variables mentioned above allows us to conclude that the functional outcome and clinical type of ICA stenosis is mainly determined by compensatory capacity of collateral circulation. Its effective functioning can able to provide adequate cerebral perfusion pressure in the compromised vascular region, despite significant reduction of arterial pressure distal to precerebral artery stenosis and prevent ischemia. In such cases, one can speak of asymptomatic cerebral hemodynamic model, which is often found in ICA thrombosis due to various etiologies. Moreover, such a high degree of collateralization of cerebral circulation allows in a number of cases to safely perform deconstructive interventions for giant ICA aneurysms, carotid cavernous fistula, resections of basal tumors incorporating ICA, without the necessity to use extracranial-to-intracranial bypass surgery (Lawton and Spetzler, 1996; Vernieri et al., 2001; Verhaeghe et al., 2003; Lubicz et al., 2010).

Collateral circulation can be visualized with digital cerebral angiography, which allows anatomically to identify the crossflow through the anterior and posterior communicating arteries, cortical-cortical, naso-orbital, and other anastomoses. But its assessment would be inadequate without measuring the velocity characteristics of cerebral blood flow and evaluating DCA. Thus, in our study, discussing the results of DCA assessment based on TCD, we certainly estimate the degree of collateral circulation compensation. And, proceeding from the received data, regardless of clinical type of stenosis, we distinguish two subgroups of patients with undisturbed and disturbed DCA, which, respectively, characterizes at least two types of functional state of collateral circulation: compensated and decompensated. It should be added that Reinhard et al. have recently studied interrelation between DCA and anatomical types of collateral circulation (Reinhard et al., 2003a). But the issue of choosing treatment modality depending on the state of DCA, and hence, collateral circulation capacity has not been discussed so far. This is the main problem raised in the present study.

There are several limitations that should be mentioned in interpreting these results. First of all, the sample size of the patients is relatively small, particular for symptomatic ICA stenosis. However, similarity of the clinical groups according to inclusion criteria presented in our study allows us to be sure in reliability of the obtained results and provides a rationale for further studies to address this question finally on a more representative sample. Besides, the number of patients according to the literature varies greatly—from 19 up to 139. The largest sample of the patients with ICA stenosis is presented by the following authors: Hu et al. (*n* = 83), Haubrich et al. (*n* = 102), Reinhard et al. (*n* = 139) (Hu et al., 1999; Reinhard et al., 2003b; Haubrich et al., 2004). At the same time, in other, important published works [Tang et al. (*n* = 21), Diehl et al. (*n* = 20), White et al. (*n* = 27), Reinhard et al. (*n* = 19), Gooskens et al. (*n* = 38)] assessing DCA the sample of the patients was almost the same as in our study (Diehl et al., 1995; White and Markus, 1997; Reinhard et al., 2001; Gooskens et al., 2003; Tang et al., 2008). Secondly, DCA was analyzed in dependence of clinical type of disease and compared only with control group of healthy volunteers. However, it is of great importance to compare the results of DCA dynamics and outcomes between the group of operated patients and the group of patients who were administered long-term isolated optimal medical therapy. Such an analysis could be the subject of a separate randomized and multicenter study, which will allow a more objective evaluation of the advantages of both treatment modalities.

Further studies are needed to be done to define prognostic value of DCA parameters for prediction of hyperperfusion complications after reconstructive surgery on stenotic carotid arteries. An incidence of cerebral hyperperfusion syndrome after CAS/CEA is quite rare—<1.9% (Moulakakis et al., 2009). Most authors associate their occurrence with impaired DCA after surgery. There were no observations of postoperative hyperperfusion complications in our study. Nevertheless, it should be noted that the bedside perioperative DCA assessment will allow more careful monitoring of cerebral hemodynamics for timely prevention of postoperative cerebral hyperperfusion syndrome that undoubtedly worsens the functional outcome of surgical treatment of patients with ICA stenosis.

It is obvious that the evaluation of the functional reserve of cerebral hemodynamics in patients with stenotic carotid lesions should become an integral part of the routine examination in order to optimize the treatment strategy. It should be admitted that the numerous studies describing DCA impairment in carotid stenosis and its restoration after surgical treatment have been published over the last three decades. Nevertheless, the present study demonstrates that even among patients with severe ICA stenosis DCA can be unaffected.

In conclusion, our study suggests that DCA non-invasively evaluated by TFA of slow oscillations of BFV and BP within the range of Mayer waves is an informative determinant of stroke risk in patients with carotid stenosis. Identification of patients with an impaired as well as with normal autoregulatory capacity may help to improve treatment strategies in the presence of both asymptomatic and symptomatic severe ICA stenosis. The risk of stroke in asymptomatic severe ICA stenosis and unimpaired DCA seems to be small. They may benefit from the optimal medical therapy more than from surgical correction of stenosis. On the contrary, patients with impaired DCA regardless the clinical course of severe stenosis are at highest risk of ischemic events, and ought to be considered as the first candidates for surgery. Surgical management of ICA stenosis contributes to restoration of impaired DCA.

## Author contributions

All authors have seen and approved the final version of the manuscript. VS is a first author who has made substantial contributions to conception and design of the article, has drafted the article, and revised it critically for important intellectual content. GA and AP has made substantial contributions to conception of the article and revised it critically for important intellectual content. AN and GP has made substantial contributions to acquisition of data, analysis, and interpretation of data. VA has made substantial contributions to analysis and interpretation of data, conception of the article, and revised it critically for important intellectual content. VI has made substantial contributions in surgical management of the patients included into the study. AS has made substantial contributions in surgical management of the patients included into the study, and revised the article for important intellectual content.

### Conflict of interest statement

The authors declare that the research was conducted in the absence of any commercial or financial relationships that could be construed as a potential conflict of interest.
